# Advance Decision Making in Bipolar: A Systematic Review

**DOI:** 10.3389/fpsyt.2020.538107

**Published:** 2020-10-16

**Authors:** Lucy A. Stephenson, Tania Gergel, Astrid Gieselmann, Matthé Scholten, Alex Ruck Keene, Larry Rifkin, Gareth Owen

**Affiliations:** ^1^ Department of Psychological Medicine, Institute of Psychiatry, Psychology and Neuroscience, King’s College London, London, United Kingdom; ^2^ Institute for Medical Ethics and History of Medicine, Ruhr University, Bochum, Germany; ^3^ Lambeth Home Treatment Team, South London and Maudsley NHS Foundation Trust, London, United Kingdom

**Keywords:** advance care planning, advance statement, psychiatric advance directives, mental capacity, human rights, mental health, mental health law

## Abstract

**Introduction:**

“Advance decision making” (ADM) refers to people planning for a future when they may lose the capacity to make decisions about treatment (decision making capacity for treatment or DMC-T). This can occur in a variety of physical and mental health scenarios. Statutory provision for ADM is likely to be introduced to mental health legislation in England and Wales, which will support planning for mental health crises. Conceptually, it may have particular utility for people with Bipolar Affective Disorder (bipolar) due to the pattern of rapid loss and then recovery of DMC-T during episodes of illness. Furthermore, ADM is recommended by clinical experts in bipolar. However, the empirical evidence base for ADM in bipolar is unclear. Therefore, a systematic review is required to collate available evidence and define future research directions.

**Methods:**

A PRISMA concordant systematic review of empirical literature on the use of ADM in bipolar.

**Results:**

We found 13 eligible articles which reported on 11 studies. Of the eligible studies 2 used a mixed methods design, 8 were quantitative descriptive studies and 1 was a randomised controlled trial. Outcomes of included studies fell into 4 categories: Interest in ADM, type of ADM preferred, barriers to completing ADM and impact of ADM. The available evidence suggests that people with bipolar are interested in engaging with ADM which is supported, collaborative and allows them to state treatment requests and refusals.

**Conclusions:**

Evidence in this area is limited. Clinicians should be aware that service users with bipolar are likely to value their support in creating ADM documents. In addition, it seems that people with bipolar may face fewer barriers and achieve greater success with ADM compared to those with other severe mental illnesses. Given the greater focus and likely demand for ADM following upcoming legal reform, further research is urgently needed to ensure available resources are most effectively targeted to achieve the best outcomes from ADM activities. This research should focus on clarifying: causal assumptions around ADM, the outcomes which are valued by key stakeholders, barriers to achieving these outcomes, stakeholder opinions on supporting ‘self-binding’ and the development and evaluation of models of ADM which are tailored for fluctuating DMC-T.

## Introduction

Bipolar affective disorder (bipolar) is a common mental disorder with prevalence estimates ranging from 1–2% of adults being diagnosed with bipolar and up to 5% of adults being impacted by a bipolar spectrum disorder. It most commonly presents in early adulthood, although for many diagnosis is delayed. Following a first episode, most recover well but about 80% relapse within five to seven years and may go on to have three or more episodes over 20 years (1). Those affected experience extreme and disabling fluctuations in mood: depression, mania and mixed states. These episodes, especially if untreated, may persist for several weeks or months. Coinciding with extreme fluctuations in mood, people with bipolar may experience fluctuations in decision making mental capacity for treatment (DMC-T), particularly during prolonged episodes of mania (2). During these periods, they may hold uncharacteristic beliefs which lead them to make uncharacteristic decisions about treatment, for example refusing medication or admission to hospital despite risks that seem obvious to professionals and loved ones. The service user’s repeated experience of these episodes and the expertise acquired around what is helpful management for them and what should be avoided lends itself to advance decision making for mental health crises.

“Advance decision making” (ADM) refers to people planning for a future when they may lose the capacity to make decisions about treatment (DMC-T).^1^ Over the past few decades support for ADM in all types of severe mental illness (SMI) has gathered momentum. Multiple jurisdictions in the UK (e.g. Scotland, Ireland), Europe (e.g. Germany, The Netherlands, Belgium), the US, Australia and India have already made formal legal provision on grounds that ADM increases individual autonomy and decreases health inequality. In England and Wales mental health ADM are likely to become more common following planned reforms to the Mental Health Act 1983. If in the form proposed by the Independent Review of the MHA 1983 these reforms will introduce “Advance Choice Documents” which offer service users the opportunity to make legally enforceable ADM documents (3).

People with bipolar may be particularly suited to using these new legal provisions, given the fluctuations in DMC-T, the typically repetitive nature of illness episodes and evidence that, compared to those living with other SMI, people with bipolar recover DMC-T more rapidly following an episode. Therefore, they may have a greater window of time in which to engage with ADM (4). Guidelines on the treatment of bipolar from the UK, (5, 6), USA (7), Australia and New Zealand (8) recommend ADM. However, these guidelines do not give empirical evidence supporting this clinical recommendation. In addition, apart from one study looking at ADM for personality disorder (9), there has been little empirical exploration of whether ADM maybe more or less effective for those with different types of SMI and whether it should be tailored accordingly. In summary, conceptually ADM may have particular utility for people with bipolar and is recommended by clinical experts. However, the empirical evidence is unclear. Therefore, a systematic review of the use of ADM in bipolar is required to collate available evidence and define future research directions.

This systematic review has the following objectives:

- Clarify the state (in terms of extent, nature and quality) of the current empirical evidence base on ADM in bipolar.
- Clarify current understanding on features of ADM that have most relevance for people with bipolar.
- Identify gaps in the current evidence base and refine future research directions.

## Method

A search was carried out, according to PRISMA guidance, which aimed to locate all relevant empirical literature on the use of mental health ADM in bipolar. Inclusion criteria were i) the paper explicitly included or discussed people with any type of bipolar and, if the sample comprised people with bipolar alongside other SMI, a sub group analysis for participants with bipolar was available; ii) the paper detailed the use of any form of advance decision making used for managing mental health crises. Exclusion criteria were i) the paper focussed on managing non-mental health scenarios e.g. physical health, dementia or end of life care; ii) the paper was in a non-English language.

### Search Strategy

The search strategy was devised using a PICOS grid, terms were selected according to our inclusion and exclusion criteria and using variations found in major known key texts. These terms were discussed and agreed among members of the research team (LS, TG, AG, MS) Due to the small evidence base on this topic and the need for a broad search only variations for terms for the “Problem” (bipolar) and “Intervention” (advance decision making) were entered into the PICOS grid. All comparators, outcomes and study designs were eligible for inclusion.

The basic search string was then tailored according to the capabilities of each database. Where available sub-headings were used and, when relevant terms were listed, options to explode subheadings were employed. Search limits were also tailored with a focus on maintaining a wide search. All years, publication types (e.g. books and papers, published and unpublished) were allowed. The searches were cross checked for re-producibility among the research team (LS and TG for initial search and LS, AG, and MS for updated search).

The texts resulting from the electronic search were compiled into a central database. Duplicates were removed. After research team members discussed and confirmed understanding of the inclusion/exclusion criteria, the titles, then abstracts, then full texts of the documents were independently searched for relevance by two team members (TG and LS) with discussions held at each stage to determine which articles should be included. Any disagreements were discussed (by TG, LS, GO) until consensus was reached.

The final list of documents for inclusions was sent to international experts in the field to request suggestions for other relevant documents that were not located in the search. The references of the included documents were also followed to ensure full coverage.

Contact with study authors of RCTs which trialled the use of ADM with people who have SMI was made to ascertain whether sub analyses of results for people with bipolar were available.

The following electronic search strategy was used for PubMed: ((((advance directive OR advance care planning[MeSH Terms])) OR (“advance decision”[Title/Abstract] OR “self-binding”[Title/Abstract] OR “psychiatric advance directive”[Title/Abstract] OR “advance agreement”[Title/Abstract] OR “advance statement”[Title/Abstract] OR “mill’s will”[Title/Abstract] OR “voluntary commitment contract”[Title/Abstract] OR “nexum contract”[Title/Abstract] OR “crisis plan”[Title/Abstract])) AND (mental disorder OR Bipolar and related disorders OR Mood disorder OR psychotic disorders OR depression OR mental health OR mentally ill persons[MeSH Terms])) NOT (dementia OR terminal care OR palliative care[MeSH Terms]).

### Data Sources

Databases were searched across all available dates: SCOPUS (1966–2019), CINAHL (1937–2019), Cochrane (1996–2019), EMBASE (1974–2019), Medline (1946-2019), PsychINFO (1806–2019) and PubMed (1865–2019). Where it was possible to specify, published and unpublished papers were included in the search filters. Initial searches were conducted in 2017 (LS, TG) and updated in 2019 (LS, AG and MS).

### Data Extraction

Standardised data extraction *pro formas* were designed by team members (TG, GO, LS). The data was extracted by one researcher (LS) and reviewed by two senior researchers (TG and GO). Like for like comparison of quality between articles was challenging due to the heterogeneous nature of the included papers. The Mixed Methods Appraisal Tool (MMAT) was used to categorise the body of literature identified, further clarifying the current state of the available evidence base (10).

### Data Analysis

Neither quantitative or qualitative meta-analysis was appropriate for the body of literature identified during the searching process. Instead, data was tabulated, arranged and synthesised in a narrative fashion (11).

## Results

### Study Selection and Characteristics

**Figure 1:**
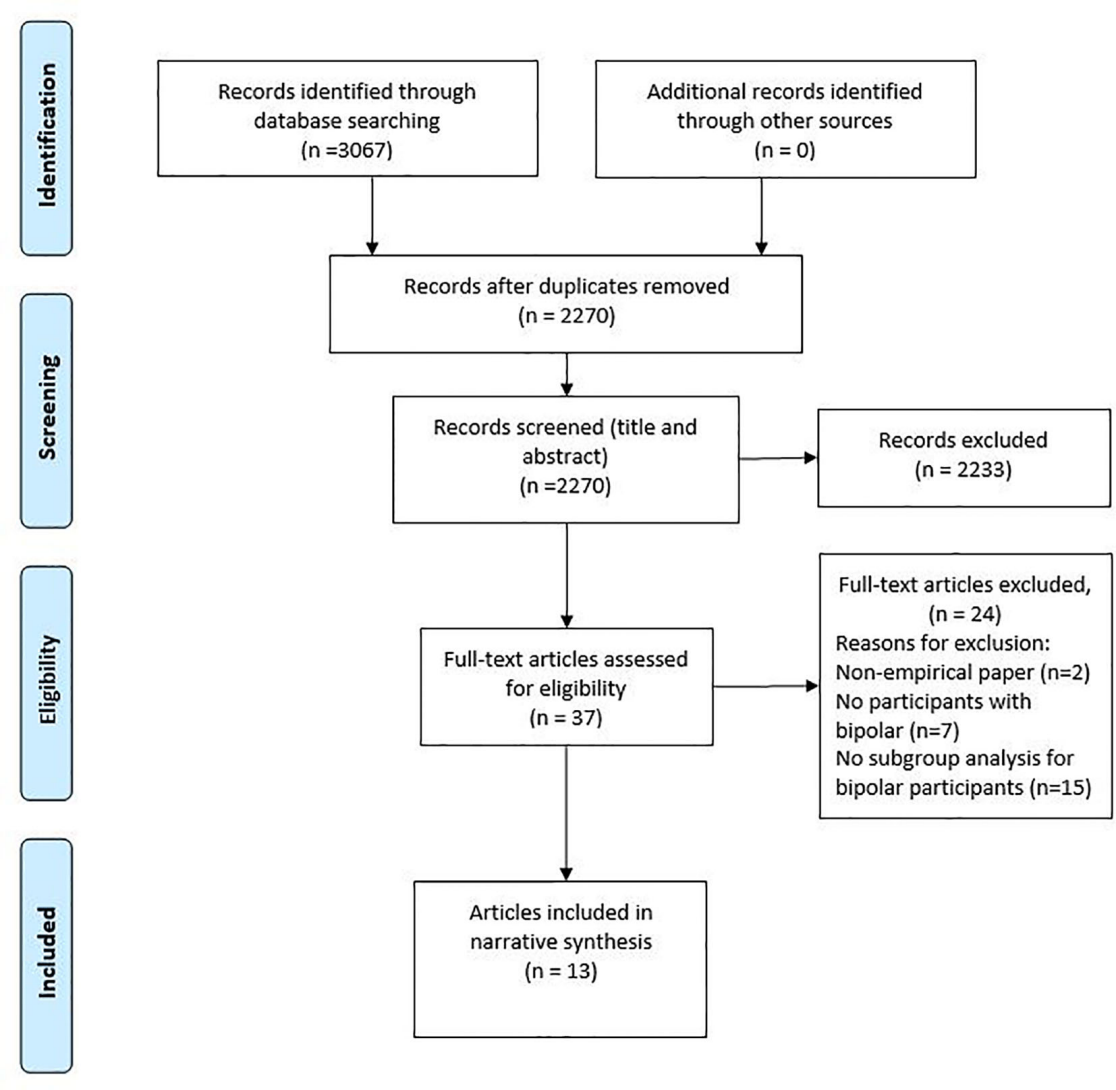
Flow diagram.

A total of 3,067 articles were identified by electronic database searches, citation searches and consultation with experts in the field. After the removal of duplicates, and title and abstract screen, a total of 37 articles remained. Following full text screening a total of 13 articles detailing 11 studies were included in the final review. **Figure 1** shows the flow diagram.

The range of study designs included 2 mixed methods studies, 7 quantitative descriptive studies and 1 randomised controlled trial. The results are summarised in **Table 1**. The focus of the studies can be grouped according to the following outcome domains for study participants with bipolar: interest in ADM, preferred type of ADM, barriers to engaging in ADM and impact of using ADM. These outcome domains were determined after a series of discussions among 3 members of the research team (LS, TG, GO) during which consensus was reached.

**Table 1:** Results.

Study number	Author/year/country/reference no	Studytype	Participants(numbers where available)	% service user participants with Bipolar	Study Objectives	Measures used	Overall conclusions	Key findings for participants with Bipolar	Outcome domains
**1**	Ambrosini et al., 2012 Canada (12)	Mixed methods	Adults with SMI known to community services Total n= 59 Bipolar n=16 Schizophrenia n=19 Depression n=24	27%	Explore factors associated with treatment preferences of individuals with SMI to complete ADM vs substitute decision maker nomination	Competence: MacCAT-CR, Autonomy: IPAS, API, Empowerment: Making Decisions Empowerment Scale, Recovery: RAS, Insight: ITAQ, Coercion: MPCS, Psychopathology: BPRS-E, Attitude toward medication: Hogan Drug Attitude Inventory, Preferences for advance directives: PAD Scale	Majority of participants chose instructional directives (76%) vs proxy directives (substitute decision maker) (24%) Factors associated with choice of substitute decision maker rather than ADM were: male gender, SCZ, greater insight into need for treatment. Qualitative interviews suggested those who chose ADM were motivated to apply their lived expertise to maintaining control over the illness. Choice of a substitute decision maker was taken by those who preferred a simpler document, who trusted others and had less knowledge about their illness	All participants with Bipolar chose ADM rather than substitute decision maker	Preferred type of ADM
**2a**	Bartlett et al., 2016 England and Wales (PARADES study) (13)	Mixed methods	Quantitative: Adults with Bipolar n=549 Psychiatrists n=650 Qualitative: Adults with Bipolar n=14 Psychiatrists n= 8	100%	Examine experience and use of advance care planning by people with Bipolar in England	Survey and semi-structured interviews	Majority of service users (82.3%) thought it very important to engage with ADM but low usage of any of MCA provisions (23.7%). Uncertainty, amongst all stakeholders, around procedures for advance care planning or local and national policy and legal frameworks	See overall conclusions	Interest in ADM Preferred type of ADM Barriers to ADM
**2b**	Morriss et al., 2017 England and Wales (PARADES study) (14)	Mixed methods	Quantitative: Adults with Bipolar Total n=544 Qualitative: Adults with Bipolar n=18	100%	Examine attitudes, knowledge and experiences of people with Bipolar to MCA provision for ADM	Survey and semi-structured interviews	High level of support for ADM amongst service users with Bipolar (74.1%) Low knowledge (36.6%) and formal use of MCA provision (16.6%) Multiple personal, interpersonal and systemic barriers to creation and use	See overall conclusions	Interest in ADM Preferred type of ADM Barriers to ADM
**2c**	Morriss et al., 2017 England and Wales (PARADES study) (15)	Quantitative results from mixed methods study	Psychiatrists Total n=650	N/A	Determine which aspects of MCA training were associated with higher or lower perceived quality of training in the view of psychiatrists, to examine whether quality of training was associated with reported attitudes or use of ADRT in people with Bipolar	Survey	MCA training is available to psychiatrists but Is not often of sufficient quality to meet the needs of those with Bipolar. Better quality training for psychiatrists on ADRTs may increase use amongst people with Bipolar MCA training rated as significantly higher quality used role play, training on ADRT and assessment of capacity. Training on the assessment of fluctuating capacity was uncommon. Higher quality training was associated with willingness to discuss ADRTs and overcoming time constraints to do so. Notable resistance to discussing ADRTs: 46.3% psychiatrists would not discuss ADRTs even if raised,	See overall conclusions	Barriers to ADM
**3**	Gowda et al., 2018 India (16)	Quantitative descriptive	Adults with SMI who were inpatients in a psychiatric hospital Total n=182 44% Bipolar and depression 47% Schizophrenia and psychotic disorders 9% Others	44%	Explore willingness to complete PADs, treatment preferences detailed in PAD and characteristics determining treatment preferences	Mini International Neuropsychiatric Interview Clinical Global Impression Scale Insight Bangalore Advance Directive Interview	Majority (96%) participants created ADM during the study Majority used ADM to: Choose a nominated representative (94%) To be treated as advised by their doctor (80%) Expressed preference for outpatient care (57%) Majority used ADM to refuse: ECT (58%) Faith healers (59%) Psychosurgery (57%).	Diagnosis of Bipolar significantly associated with using ADM for treatment requests	Preferred type of ADM
**4**	Hindley et al., 2019 England & Wales (17)	Quantitative descriptive	Adults with Bipolar Total n=182	100%	Compare experiences of ADM with preferences for ADM amongst people with Bipolar Describe experiences of using ADM in crisis Explore attitudes towards ADM to Identify demographic and clinical variables associated with interest in ADM	Survey	Majority of service user participants (88%) would like to engage with ADM but majority (64%) had not done so. Majority (66%) of service users who had engaged in ADM had not used legal provision. Of those who used legal provision advance statements were most common (17%) Most (70%) would like to collaborate with a psychiatrist to produce ADM document Preferred ADM content was: Nominating proxy decision maker (89%) Preferred crisis contacts (87%) Financial arrangements (77%) Requests for treatment location (70%) Requests for medication (69%) Requests for hospitalisation (68%) Specific medication refusals (68%) ECT refusal (63%) Discharge planning (61%)	See overall conclusions	Interest in ADM Preferred type of ADM Barriers to ADM Impact of ADM
**5**	Khazaal et al., 2008 Switzerland (18)	Quantitative descriptive	Adults who had experience of compulsory detention Total n=20 Bipolar n=17 Schizoaffective n=3	85%	Test feasibility of Advance Directives Based on Cognitive Therapy	Content and feasibility of ADM documents	A psychological intervention combining principles from CBT and MI was feasible as a method of supporting people with Bipolar to create ADM documents	See overall conclusions	Preferred type of ADM
**6**	Khazaal et al., 2009 Switzerland (19)	Quantitative descriptive	Adults with Bipolar who had experience of compulsory detention Total n=18	100%	Evaluate the impact of Advance Directives Based on Cognitive Therapy	Pre-test, post-test: hospitalisations, compulsory admissions, days spend in seclusion, length of admission	Significant reduction in hospitalisations, compulsory admissions and length of stay	See overall conclusions	Preferred type of ADM Impact of ADM
**7**	Srebnik et al., 2003 USA (20)	Quantitative descriptive	Adults known to outpatient psychiatric services with at least 2 crises in previous 2 years requiring emergency outpatient or inpatient treatment Total n=303 Bipolar n=77 Schizophrenia n=155 Depression n=61	25%	Determine rates of interest in ADM (PADs) and factors associated with interest in high users of crises and hospital services	Case manager inquiry about interest, structured questions	The majority of participants with SMI (53%) were interested in ADM, attitudes of clinicians strongly influenced interest in the service users	Bipolar not more significantly associated with interest in ADM compared to those with other diagnoses	Interest in ADM
**8**	Srebnik et al., 2004 USA (21)	Quantitative descriptive	Adults known to outpatient psychiatric services with at least 2 crises in previous 2 years requiring emergency outpatient or inpatient treatment Total n=80 Bipolar n=20 Schizophrenia n=41 Depression n=18 PTSD n=1	25%	Describe initial properties and psychometric data on competence of people with SMI to complete ADM documents (PADs) using CAT-PAD (Competence Assessment Tool for Psychiatric Advance Directives)	AD-maker competence questions Psychiatric Symptoms Assessment Scale Problem severity summary	People with SMI are likely to have capacity to complete PADs, CAT-PAD is a reasonable instrument to use to asses capacity to complete ADM	Participants with Bipolar had significantly higher total CAT-PAD scores (suggesting higher competence to complete ADM) than participants with Schizophrenia	Barriers to ADM
**9**	Srebnik et al., 2007 USA (22)	Quantitative descriptive	Adults known to psychiatric services who had made a Psychiatric Advance Directive Total n=106 Bipolar n=28 Schizophrenia n=55 Depression n=27	26%	Examine the rate and predictors of receiving care that is consistent with advance directive instruction	Directive user interview Chart review Electronic patient information Problem Severity Summary Psychiatric Symptom Assessment Scale, Directive utility ratings	The majority of crisis care (67%) was consistent with ADM instructions	Service users with Bipolar experienced care that was most consistent with advance directive instructions	Impact of ADM
**10**	Swartz et al., 2006 USA (23)	Quantitative descriptive	Adults who participated in randomised trial of a facilitated Psychiatric Advance Directive intervention Total n=131 Bipolar 27% Schizophrenia 59% Depression with psychotic features 14%	27%	Explore service user preferences for ADM components and factors governing these preferences	Questionnaire about ADM preferences: Irrevocability during a crisis Treatment refusals Treatment requests Surrogate decision making	Diverse ADM preferences across the sample, majority’s preferences motivated by wish to maintain continuity of care by requesting treatment as advised by their doctor	Participants with Bipolar had lower preference for irrevocable ADM	Preferred type of ADM
**11**	Van Dorn et al., 2008 USA (24)	Randomised controlled trial	Adults with severe mental illness known to community psychiatric services who participated in randomised trial of a facilitated Psychiatric Advance Directive intervention Total n=469 Bipolar 28% Schizophrenia 59% Depression with psychotic features 16%	28%	Explore potential barriers related to ADM document completion	Questionnaire on ADM barriers designed by research team, Working Alliance Inventory, Mental Health Statistics Improvement Program Consumer Survey Index of Treatment Satisfaction Number of monthly outpatient visits Brief Psychiatric Rating Scale,	At follow up barriers to ADM document completion were significantly lower in the experimental group	Participants with Bipolar reported significantly fewer barriers to ADM document completion	Barriers to ADM

### Synthesized Findings

#### Interest in ADM Among People With Bipolar

All the studies included in this review confirmed interest among participants with bipolar.

Bartlett et al. (13) and Morriss et al. (14) report on a survey of the opinions of over 500 adults with bipolar on their attitudes towards ADM and their knowledge and use of the Mental Capacity Act 2005 (MCA) provision supporting ADM in England and Wales. In a later study, Hindley et al. (11) surveyed over 900 adults with bipolar. The results of both these studies suggested a high level of demand for ADM but low levels of use, and particularly low levels of use of the available provision under the MCA.

Srebnik et al. (20) conducted a study in the US assessing interest in ADM with over 300 adults with SMI by recruiting service users case managers to give service users information about key concepts in creating ADM documents and then ask if they would like to create one. If service users were interested the study team asked them an open ended question about the reason for their interest and recorded the verbatim responses. They found the majority (53%) were interested in ADM, those with bipolar were not significantly more or less interested than those with other SMI.

#### Preferred Type of ADM

Ambrosini et al. (12) conducted a mixed methods study which involved interviewing and giving participants with SMI questionnaires exploring domains including autonomy, empowerment, recovery, experience of coercion and attitude towards treatment before and after they were offered the opportunity to complete either an instructional or a proxy directive (i.e. nominate a substitute decision maker). The impact of these factors on preferences for either form of directive were then explored. Of the total sample, the majority (76%) chose instructional directives. Key motivating factors for this choice were that participants wished to apply their lived expertise to maintaining control over the illness. Of note, all participants with bipolar chose an instructional directive.

The two surveys exploring how many of their total sample of people with bipolar had actually used formal provision for ADM under the MCA (13, 14, 17) both found that it was more common for people with bipolar to make Advance Statements with treatment *requests* [around 11% (13, 14) and 17% (17)] rather than Advance Decisions to Refuse Treatment i.e. treatment *refusals* [around 10% (13, 14) and 6% (17)]. Morriss et al. (14) found that use of this formal ADM provision was more likely in those who had a care coordinator or belonged to an NHS support group.

Hindley et al. (17) also asked participants about their aspirations for making ADM even if they had not actually made any formal ADM documents. Most (70%) would like to collaborate with a psychiatrist to make an ADM document but only 14% had been able to achieve this. The highest majority stated they would use ADM to document who they would like to make decisions if they were unable to do so (89%) followed by preferred crisis contacts (87%) and then financial arrangements (77%), requests for treatment location (70%), medication requests (69%), hospitalisation requests (68%) medication refusals (68%), ECT refusal (63%) and discharge planning (61%). The majority of respondents (69%) were in favour of having a “self-binding” component as part of their ADM document which would support an advance request for compulsory treatment whilst acknowledging that at the time treatment was required they would be likely to refuse. This interest was significantly associated with having trust in health professionals.

Gowda et al. (16) explored service user preferences for ADM among 182 (44% bipolar) adults in an inpatient Indian setting, where the Mental Health Care Act 2017 has recently been passed. This contains statutory provision for people to make mental health advance directives. This study also demonstrated that a diagnosis of bipolar was significantly associated with a wish to use ADM to make treatment requests.

Swartz et al. (23) sought to explore service user preferences for particular features of ADM among 131 adults with SMI (27% had bipolar). Preference options for ADM included: irrevocability during a crisis, treatment refusals, treatment requests and appointing a proxy decision maker. Compared to those with other diagnoses, they found that participants with bipolar had a lower preference for irrevocable ADM. This is interesting given the finding by Hindley et al. (17) that the idea of “self-binding” appealed to the majority of participants.

Khazaal et al. (18, 19), working under the Swiss provision for ADM, developed and piloted a process of engaging service users with bipolar in creating ADM. This was based on principles drawn from Cognitive Behavioural Therapy and Motivational Interviewing. Participants received an average of 4 sessions with a trained professional which began whilst they were an inpatient in hospital and understood to be in an “ambivalent” phase and therefore open to discussing the pros and cons of illness manifestations and treatments. The session topics included: legal information about ADM, exploration of service user’s cognitive representation of their illness, relapse indicators, concerns about previous treatments, long term prevention strategies, creating ADM document, application of ADM in future crises and review of ADM. The service user then created an ADM document independently. The authors piloted this intervention with 20 services users with bipolar and concluded that following the intervention all wrote clinically feasible ADM documents.

#### Barriers to ADM

The two surveys of people with bipolar identified barriers at a personal, interpersonal and systemic level to ADM. These included: lack of service user and clinician knowledge about ADM (13–15, 17), lack of support in creating ADM (13, 14, 17), unrealistic expectations (14), accessibility issues (13, 14, 17), and ADM being ignored by mental health services (13, 14, 17).

However, other studies suggested that although these barriers might exist, people with bipolar may, overall, face fewer obstacles than those with other SMI. Van Dorn et al. (24) found that of participants in a RCT of facilitated ADM documents (PADs) those with bipolar (rather than Schizophrenia or Depression) reported significantly fewer barriers to document completion. Srebnik et al. (21) tested an instrument (CAT- PAD) designed to assess competence to engage in ADM with a community sample (i.e. well enough not to require inpatient treatment at the time of testing) of people with SMI. They found that participants with bipolar or depression had significantly higher total scores, signalling higher levels of competence compared to participants with other SMI.

#### Impact of ADM

Hindley et al. (17) reported that of the minority (26%) of survey respondents who had experience of using their ADM document in a crisis, half (50%) felt their document was respected by staff. The majority felt they recovered faster (60%), had an improved experience of health services (60%) and were generally happy with how their document had been used (55%). In terms of the potential of ADM to help avoid harms, participants saw greater potential for the use of ADM to reduce social (44%) and physical (34%) harms compared to financial harms e.g. overspending money when unwell (23%).

Srebnik et al. (22) explored the extent to which ADM preferences were followed in a crisis. They found that service users with bipolar (compared to those with other SMI) experienced care that was significantly more consistent (p=0.002).

Khazaal et al. (19) explored the impact of ADM on coercive interventions and found that people who had participated in their ADM focussed intervention had significantly reduced hospitalisations, compulsory admissions and length of stay.

## Discussion

### Summary of Main Findings

This first systematic review of the literature on ADM for bipolar aimed to: establish the state of the current evidence base, clarify key features of ADM which are relevant to people with bipolar, identify gaps in the evidence base, and refine future research directions. We found a small evidence base which is at an early stage of developments and comprises largely heterogeneous observational study designs exploring a range of ADM types. Given the current state of the evidence it is not possible to draw any firm conclusions about the effectiveness of ADM for people with bipolar in achieving outcomes stakeholders desire. However, the available research appears to most strongly confirm a high level of interest in ADM among people with bipolar. ADM features that seem to be important to this group include support for collaboratively made, instructional directives which facilitate treatment *requests* as well as refusals. There is some inconsistency between studies about whether or not people with bipolar are likely to support “self-binding”, which merits further exploration. Several studies identify multiple and significant barriers to ADM for bipolar and there is a far greater focus on factors which are barriers, compared to those that facilitate ADM. However, it may be that people with bipolar face comparatively fewer barriers. ADM appears to hold promise for people with bipolar in reducing coercion and improving the experience of using mental health services in crises. Again, this is not a guaranteed outcome, but it seems that those with bipolar may have better chances of making and successfully using ADM documents than those living with other SMI for reasons that are, as yet, unclear. Gaps in the current evidence base include: understanding outcomes that service users with bipolar wish to achieve using ADM, understanding causal mechanisms and features of ADM that allow these outcomes to be achieved, clarifying service user opinion on self-binding ADM and developing a model of ADM and materials tailored for people with bipolar. Developing models of collaborative ADM could be helpfully informed by existing research on shared decision making (SDM) in mental health.

### Limitations

Limitations of this review include the inherent difficulties of evaluating and synthesising results from a range of study types many of which are pilot-like. It is of note that none of the RCT studies and meta-analyses which explore outcomes for ADM in SMI (25–31) provide sub analyses according to individual diagnoses. Instead, a broad division is frequently made between psychotic and affective disorders. This is of concern given the differences in psychopathology between various SMI and the varying patterns of assistance individuals with bipolar are likely to require from health services (32). In addition, the included studies do not specify whether participants had a diagnosis of type 1 or type 2 bipolar. It is likely that a range of severity is represented in this review as the included two service user surveys relied on self-report diagnosis by service users with any level of mental health input (13, 14, 17). Whereas the other studies recruited service users with a diagnosis of bipolar who were actively in touch with specialist psychiatric services (requiring outpatient visits/emergency contacts/hospital admissions) (12, 16, 18, 19, 21–24).

Therefore, given the studies that were eligible for inclusion only a low level of confidence in the results is possible. However, a strength of this review is the comprehensive search and wide inclusion criteria which aimed to establish the state of the evidence base.

### ADM in Bipolar Compared to ADM in General SMI

The results of this review focussing on ADM in bipolar are largely consistent with the literature on ADM in general SMI. This literature also suggests that ADM is supported by service users (33) and that service users tend to produce clinically feasible documents (34, 35). Despite this enthusiasm, there are often low levels of uptake (33).

A key difference is that in the wider literature on ADM in SMI there have been larger scale RCT studies of the outcomes of using ADM. The purpose and primary outcome of ADM in these RCT studies is generally considered to be avoiding coercion, particularly in the form of compulsory inpatient admissions. Studies on this topic have had conflicting results, although meta-analyses suggest that overall ADM reduces coercion for people with SMI. Given that people with bipolar may face comparatively fewer barriers to completing ADM (24) and that their ADM preferences may be more likely to be followed (22) it could be hypothesised that this group is more likely to achieve success.

### ADM Outcomes

It remains unclear exactly what service users and professionals believe ADM are for i.e. which outcomes we might expect and want from their implementation. One outcome which has been empirically explored with people who have bipolar is the reduction in coercion (largely distilled into meaning reduction in the number of compulsory admissions) from mental health services. Related to this is the emergence of the subjective experience in the reduction of coercion or increase in self-determination as an outcome in itself.

Among the wider literature on ADM for SMI, RCT studies have used the reduction or avoidance of hospital admission as the primary outcome (25–28, 31). Avoiding admission and coercion has been understood to be crucial in increasing self-determination and therefore affording people with SMI equal rights. Redressing inequality in this area is a key concept of the UN Convention on the Rights of Person with Disabilities (36).

However, this perspective that a reduction in coercion leads to increased self-determination is not without challenge, particularly in the bipolar population. In their survey, Hindley et al. found a third of respondents with bipolar would use an ADM document to request admission. In another survey of over 500 service users with experience of mania and carers, most respondents (the majority of whom were service users) supported the use of coercion when unwell (37). Gergel and Owen (4) argue that earlier admission, even if facilitated through coercion, will ultimately be more effective in promoting self-determination. The person is thus empowered to harness the strength of the available legal provision and wield it as they see appropriate in order to gain more control of their illness and the consequences that may have occurred during episodes of mania/depression in the past.

Therefore, it seems prudent to challenge the assumption that ADM may be automatically judged as operating successfully if admissions are reduced through their use. If an ADM document expresses the choices of an individual it is likely that different people and groups will wish to achieve or remedy different things. Future research should focus on the outcome of harm avoidance in a broader sense, and as defined by the service user. For some, who experience compulsory admission as the main harm resulting from a crisis, this may mean early intervention to prevent admission, for others this may mean early admission to avoid other harms e.g. social or financial.

Implementation of ADM has jumped ahead of the research on outcomes. One reason for this is the ethical imperative to maximise an individual’s autonomy, particularly when autonomy is compromised. For those with bipolar, autonomy may be threatened on multiple levels: firstly, they may experience periods of illness as inauthentic i.e. not arising from their true sense of self; secondly, during periods of illness an individual may experience a loss of self-control, engaging in behaviours they would never normally consider; thirdly, attempts at illness management may elicit coercive measures from other parties such as family, friends or health professionals. Given the purported centrality of autonomy as a stated driver for implementing ADM (38) there is a strong argument for exploring whether using ADM increases service users’ sense of control over their illness and treatment as a primary outcome.

### Future Research and Clinical Implications

The Medical Research Council (MRC) defines complex interventions as those that: have “a number of interactions between components within the experimental and control conditions”, require “a number and difficulty of behaviours required by those delivering or receiving the intervention”, target “a number of groups or organisational levels”, have “a number and variability of outcomes” and require “flexibility or tailoring of the intervention” (39). This is undoubtedly the case for ADM in bipolar. Particular dimensions of complexity include: Creating an ADM document is a highly individualised process (40), successfully implementing ADM is likely to depend on an organised response from several services e.g. mental health teams, Accident and Emergency departments, specialist social workers, inpatient wards and the desired outcomes are (as yet) unclear and likely to include subjective measures.

This review has confirmed that more research on ADM as a complex intervention is required. MRC guidelines specify that developing and evaluating a complex intervention usually involves developing a conceptual model, followed by feasibility testing, evaluation and then implementation. Evaluation commonly takes the form of randomised control trials to determine the effectiveness of an intervention and therefore justify larger scale implementation. However, in multiple international jurisdictions (soon to include England and Wales following their government’s acceptance of the recommendation of the Independent Review of the MHA) ADM has already been introduced largely on the basis of stakeholder consensus and strong ethical and human rights grounds. Therefore, the important outstanding research questions for jurisdictions which already have statutory provision and those who are considering introducing it are less around examining effectiveness to build a case for introducing ADM and more around achieving high quality implementation and identifying potential harms. This will involve refining understanding of enabling conditions (including training and materials), key features of process and desired outcomes. For jurisdictions where legislation is in force further research will be essential to determine whether legal changes have been effective in achieving valued outcomes for stakeholders.

Future research directions could include the following:

1. Clarifying causal assumptions about the potential of ADM to achieve positive and negative outcomes for people with bipolar.
2. Determining which ADM outcomes are valued by key stakeholders and the factors which determines these values.
3. Greater understanding of facilitators as well as barriers to successful creation and use of ADM. This study suggests these barriers include lack of: clinician knowledge and training about ADM, service user knowledge about ADM, support for making ADM. Little is known about facilitators.
4. Development of clinician training on ADM.
5. Understanding of the opinions of stakeholders on supporting ‘self-binding’ in ADM for bipolar.
6. Development of a model of ADM in the form of a template document and supporting materials tailored for bipolar. This study suggests that such a model should include a supportive environment, collaboration with psychiatrists, support for treatment requests and refusals and have an option for “self-binding”.
7. A prospective study piloting these materials with a focus on process evaluation.
8. Examination of efficacy of legislative change in supporting ADM to achieve valued outcomes.

## Conclusions

Clinicians working with people who have bipolar should be aware that service users are likely to be interested in ADM and would highly value their input in creating them. For service users, ADM may offer a tool to redress the loss of autonomy experienced during periods of illness and compulsory treatment and apply their lived expertise. For services, ADM documents offer the potential to increase clinical efficiency around decision making in crises plus targeted use of intensive interventions (such as inpatient admissions). However, the process of drafting and applying ADM documents demands upstream resource investment to gain these downstream rewards. Legal reform may be important in stimulating much needed resource provision supporting ADM. Further research focussed on understanding the complexities of making and using ADM documents and the impact of ADM across time is required. This research will help to ensure resources are appropriately directed to supporting high quality ADM which is likely to have the greatest potential to achieve desired outcomes for all stakeholders.

## Author Contributions 

LS contributed to the design of the search strategy, conducted the initial and updated searches, contributed to team discussions around analysis of the findings, and wrote the first draft of the paper. TG contributed to the design of the search strategy, conducted the initial searches, contributed to team discussions around analysis of the findings, and edited drafts of the paper.AG and MS contributed to the design of the search strategy and conducted updated searches. ARK contributed to team discussions around analysis of the findings and edited drafts of the paper. LR contributed to team discussions around analysis of the findings and edited drafts of the paper. GO contributed to the design of the search strategy, contributed to team discussions around analysis of the findings, and edited drafts of the paper. All authors contributed to the article and approved the submitted version.

## Funding

MS reports funding from the German Federal Ministry of Education and Research (SALUS; grant no. 01GP1792). This research was funded by Wellcome Grant no: 203376.

## Conflict of Interest

The authors declare that the research was conducted in the absence of any commercial or financial relationships that could be construed as a potential conflict of interest.

## Footnotes

1 Advance decision making can relate to other decisions e.g. finances.
